# Vascular Access Outcomes in Patients with Autosomal Dominant Polycystic Kidney Disease

**DOI:** 10.34067/KID.0000000000000453

**Published:** 2024-05-01

**Authors:** Suzanne L. Laboyrie, Maria K. Svensson, Sabine Josemans, Birgitta Sigvant, Joris I. Rotmans, Gunilla Welander

**Affiliations:** 1 Department of Internal Medicine, Leiden University Medical Centre, Leiden, The Netherlands; 2 Department of Medical Sciences Renal Medicine, Uppsala University, Uppsala, Sweden; 3 Uppsala Clinical Research Centre, Uppsala University, Uppsala, Sweden; 4 Department of Surgical Sciences, Center of Clinical Research, Uppsala University, Uppsala, Sweden; 5 Center of Clinical Research, Region Värmland, Sweden

**Keywords:** ADPKD, arteriovenous access, arteriovenous fistula, arteriovenous graft, arteriovenous shunt, dialysis, dialysis access, vascular access

## Abstract

**Key Points:**

- More patients with autosomal dominant polycystic kidney disease received their first intervention to re-establish vascular access patency.
- Patients with autosomal dominant polycystic kidney disease do not require differential monitoring and treatment of hemodialysis vascular access.

**Background:**

Autosomal dominant polycystic kidney disease (ADPKD) is a leading hereditary cause of ESKD, often using hemodialysis as a form of RRT. Patients with ADPKD may also present with extrarenal manifestations, including arterial aneurysms. The gold standard for hemodialysis access is an arteriovenous vascular access (VA), such as arteriovenous fistulas (AVFs) or arteriovenous grafts (AVGs). However, limitations, such as low VA flow and inadequate AVF outward remodeling, affect VA utilization. This study aimed to explore whether ADPKD affects patency rates of AVFs/AVGs in comparison with other underlying ESKD causes.

**Methods:**

We conducted a retrospective cohort study using data from the Swedish Renal Registry from 2011 to 2020, with follow-up until 2022. We included 496 patients with ADPKD and 4321 propensity score–matched controls. VA patency rates of patients with ADPKD were compared with those of non-ADPKD patients using Kaplan–Meier survival curves and Mantel–Cox log-rank test. Interventions to maintain or restore patency were also analyzed.

**Results:**

Patients with ADPKD constituted 8.0% of all patients, with a higher proportion in the pre-ESKD phase during VA creation (51.6% versus 40.6%). No significant differences were observed in primary, postcannulation primary, secondary, or functional patency between patients with ADPKD and non-ADPKD patients. However, more VAs were ligated in patients with ADPKD (10.5% versus 7.7%, *P* = 0.03), and they underwent more first interventions to re-establish flow (49.4% versus 41.9%, *P* = 0.02).

**Conclusions:**

These findings suggest that AVF/AVG patency remains comparable in patients with ESKD with or without ADPKD, and VA monitoring and treatment strategies for patients with ADPKD should align with those for individuals with other ESKD causes.

## Visual Abstract

**Figure:**
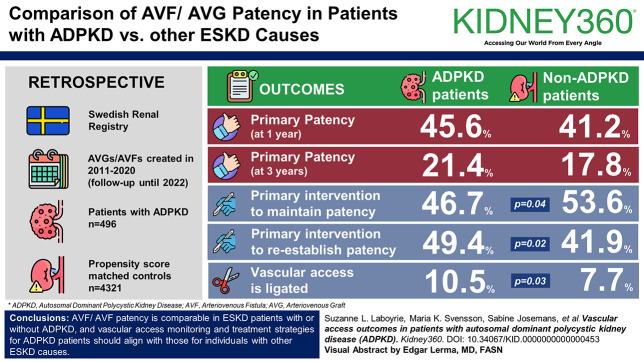

## Introduction

Patients with ESKD require RRT. Seventy percent of patients with ESKD are treated with hemodialysis,^1^ for which a well-functioning vascular access (VA) is needed. The National Kidney Foundation's Kidney Disease Outcomes Quality Initiative guideline recommends an arteriovenous fistula (AVF) or arteriovenous graft (AVG) as the preferred type of VA.^2^ However, low flow due to stenosis or occlusion is a major complication, limiting the use of AVFs and AVGs for hemodialysis. Once the VA can be used for hemodialysis, luminal narrowing through intimal hyperplasia and complications, such as steal syndrome and aneurysm formation, still pose challenges to maintain access patency. These complications require surgical and endovascular interventions and entail a large burden on both the patient and society. Therefore, understanding how individual patient characteristics can affect VA outcomes is of utmost importance.

There are many risk factors influencing VA patency, such as sex, age, comorbidities, and race.^3–6^ However, whether the underlying cause of renal failure affects access outcome is often overlooked. Approximately 9% of European patients with ESKD have autosomal dominant polycystic kidney disease (ADPKD) as their underlying disease.^7^ Most patients with ADPKD on hemodialysis use an AVF as VA.^8^ Patients with ADPKD have an increased incidence of aneurysm formation, mainly intracranial and abdominal,^9–12^ indicating altered vascular remodeling and an increase in outward remodeling in the vessels, but inducing complications in the long term. Increased AVF dilatation has also been observed in patients with ADPKD^13^ with a higher frequency of AVF aneurysms compared with other patients with ESKD.^14^ Lee *et al.*^15^ recently showed that Taiwanese patients with ADPKD have a higher risk of AVF and AVG dysfunction in the period between the first and tenth year after VA creation when compared with other patients on hemodialysis. They did not report on the effect of this increase in dysfunction or whether it affected patency of the VAs. To evaluate the prognosis of AVFs/AVGs in European patients with ADPKD, who may have a different genetic background,^16^ and if ADPKD affects patency rates, we performed a retrospective cohort study using the Swedish Renal Registry, encompassing all patients with kidney disease nationwide.

## Methods

### Study Design and Patient Selection

The Swedish National Quality Registry for Renal Failure (SRR) is a web-based registry of patients with treated kidney disease, with national coverage. The dataset is a validated resource for current VA care, with high external and internal validity.^17^ This study was approved by the Regional Ethical Review Board in Uppsala (Dnr 2017/047 and Dnr 2022-07164-02).

We included all patients who received an AVF or AVG between 2011 and 2020, with a follow-up of at least 2 years. Analyses were limited to the first AVF/AVG that was created during our inclusion period. Underage individuals (younger than 18 years) were excluded from our dataset, as well as VAs that were created in the lower limbs or deemed as technical failures. Technical failure was defined as abandonment within 3 days after creation, on the basis of the definition of immediate VA failure described by Lee *et al*.^18^ Patients who received an AVF/AVG but never proceeded to hemodialysis treatment because of receiving a donor kidney or not progressing to RRT at all were also excluded from analysis. Second, if patients were already on dialysis and never had a first cannulation nor received an intervention, we excluded them from our dataset because this might indicate continued use of a previous VA—such as a central venous catheter—for hemodialysis. To avoid confounding by indication, AVFs/AVGs that received an intervention or had a first cannulation but were not used for hemodialysis were included. This resulted in inclusion of 496 patients with ADPKD and 5675 patients without ADPKD who received an AVF or AVG. The ADPKD diagnosis in Sweden is made through radiologic imaging and the patient's family history. While planning for VA surgery, preoperative investigational mapping is performed using duplex ultrasound, with a general cutoff value of 2 mm in diameter. The individual surgeon, however, decides whether a patient is eligible. This is influenced by the health of the vessels, such as the degree of arterial atherosclerosis and distensibility of the vein.

### Definition of End Points

First cannulation is cannulation of the VA with one needle to assess whether the VA can be punctured, and a functional start of the AVF/AVG indicates three successive hemodialysis sessions with two-needle cannulation. Assisted maturation indicates a functional start that was achieved after an intervention. Maturation without an intervention was defined as an intervention-free functional start.

Primary patency was defined as intervention-free VA survival and spans from the date of VA creation until the first intervention, abandonment, end of the observation period, or censoring, whichever occurred first. Postcannulation primary patency was the period from the first cannulation of the VA until the first intervention, abandonment, end of the observation period, or censoring. Secondary patency (cumulative survival of the VA) is defined as the time of VA creation until abandonment, end of the observation period, or censoring, including all performed interventions. Functional patency spanned from the date of first cannulation until abandonment, end of the observation period, or censoring, including all performed interventions. Survival curves were plotted until 100 months because of low number of people at risk in the last months of follow-up (100–140 months).

Abandonment indicated functional loss of the VA, due to occlusion or ligation, the patient refusing use, or creation of a new VA. Censoring occurred when the patient was lost to follow-up because of emigration or death or when the patient switched to a different RRT modality, such as donor kidney transplantation or peritoneal dialysis.

First interventions performed were categorized as interventions to maintain or re-establish flow. Interventions to maintain flow included percutaneous transluminal angioplasty, ligation of collateral veins, flow reductional interventions, and transpositions for VAs that were not a two-stage brachiobasilic AVF. Interventions to re-establish flow included thrombectomies, anastomosis revisions, and thrombolysis.

### Statistical Analyses

The information that was retrieved from the SRR was compiled from the SRR datasets on CKD, VA and patient characteristics. This was combined into one dataset matched on a unique VA ID number using Python.

Baseline characteristics were summarized as mean±SD for continuous variables and compared using the *t* test, and frequency (percentage) for categorical variables, such as types of primary interventions, were analyzed using the chi-square test. The ADPKD group (496 patients) versus non-ADPKD controls (6171 patients) in our dataset had different baseline characteristics (Table 1) and were, therefore, matched on using propensity score matching on the basis of the covariate’s sex, age, location of the VA, and phase of kidney failure (having a transplant, being in chronic kidney failure, or receiving dialysis). Nearest neighbor matching in a 1:10 ratio was performed using the MatchIt package^19^ in R-studio, with a 0.1 caliper. Histograms of the propensity scores of the two groups before and after matching are presented in Supplemental Figure 1.

**Table 1:** Baseline characteristics of Swedish patients who received an arteriovenous fistula or arteriovenous graft in the arm between 2011 and 2020 in Sweden

Characteristic	Non-ADPKD Patients (*n*=5675)	Patients with ADPKD (*n*=496)	*P* Value
Age, mean years (SD)	63.9 (14.7)	61.2 (12.2)	0.001
**Sex, No. (%)**			0.001
Male	3844 (67.7)	274 (55.2)	
Female	1831 (32.3)	222 (44.8)	
**Anticoagulation, No. (%)**			0.42
Yes	1006 (17.7)	81 (16.3)	
No	1153 (20.3)	113 (22.8)	
Unknown	3516 (62.0)	302 (60.9)	
**Cause of renal failure, No. (%)**			0.001
ADPKD	0 (0.0)	496 (100)	
GN	896 (15.8)		
Pyelonephritis	191 (3.4)		
Hypertension	1049 (18.5)		
Renovascular disease	31 (0.5)		
Diabetic nephropathy	1643 (29.0)		
Unknown uremia	636 (11.2)		
Other	1229 (21.7)		
**Type of VA, No. (%)**			0.204
Radiocephalic AVF	3485 (61.4)	303 (61.1)	
Brachiocephalic AVF	1209 (21.3)	92 (18.5)	
Brachiobasilic AVF	194 (3.4)	22 (4.4)	
Forearm AVG	61 (1.1)	5 (1.0)	
Upper arm graft	601 (10.6)	56 (11.3)	
Forearm AVF other	125 (2.2)	18 (3.6)	
**Phase at VA surgery, No. (%)**			0.001
Preemptive	2305 (40.6)	256 (51.6)	
On dialysis	3004 (52.9)	190 (38.3)	
Kidney transplant	198 (3.5)	35 (7.1)	
Unknown	168 (3.0)	15 (3.0)	
**Comorbidities, No. (%)**			
Hematologic malignancy	122 (2.1)	1 (0.2)	0.12
Skin malignancy	47 (0.8)	1 (0.2)	0.31
Other malignancy	502 (8.8)	21 (4.2)	0.002
Diabetes mellitus	2202 (38.8)	30 (6.0)	0.001
Ischemic heart disease	910 (16.0)	31 (6.3)	0.001
Treated for hypertension	3586 (63.2)	306 (61.7)	0.8
Cardiovascular disease	398 (7.0)	32 (6.5)	0.89
Peripheral vascular disease	392 (6.9)	10 (2.0)	0.001

The list of causes of renal failure listed as “other” is presented in Supplemental Table 1. Dialysis treatment encompasses both peritoneal dialysis and hemodialysis. ADPKD, autosomal dominant polycystic kidney disease; AVF, arteriovenous fistula; AVG, arteriovenous graft; VA, vascular access.

Primary, postcannulation primary, secondary, and functional patency rates were presented as survival analyses using Kaplan–Meier curves and Mantel–Cox log rank test. IBM SPSS Statistics version 29 was used for all analyses (IBM Corp., Armonk, NY).

## Results

### Characteristics of Patients Receiving an Arteriovenous Access

**Figure 1:**
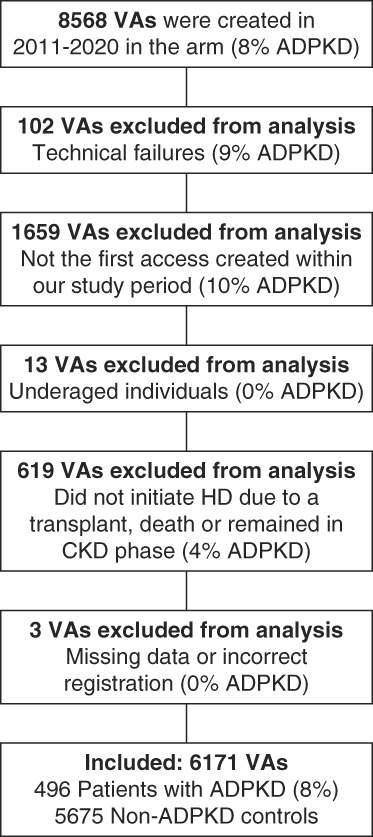
**Study flow chart.** Overview of the process of establishing the complete dataset of patients receiving an AVF or AVG between 2011 and 2020. The percentage of individuals with ADPKD in each flow chart box indicate the percentage of the excluded AVFs/AVGs per exclusion criterion that belong to patients with ADPKD. ADPKD, autosomal dominant polycystic kidney disease; AVF, arteriovenous fistula; AVG, arteriovenous graft; VA, vascular access: arteriovenous fistulas and grafts.

We retrieved all 8568 AVFs/AVGs from the SRR that were created in the arm between 2011 and 2020. After excluding VAs according to the defined exclusion criteria (Figure 1), 6171 patients were included, of which 8% with ADPKD (*n*=496) and 5675 patients with other kidney diseases (non-ADPKD controls). The baseline characteristics of the two groups are presented in Table 1. There were more female patients (44.8% versus 32.3%, *P* = 0.001) and patients preemptively receiving an VA (51.6% versus 41.6%) in the ADPKD group, whereas most of the control group (non-ADPKD) received dialysis treatment when undergoing VA surgery (38.3% of patients with ADPKD versus 52.5% non-ADPKD). A majority of the VAs in our cohort was created preemptively, but only 55 individuals (0.9%) who received an AVG/AVF did not progress to dialysis treatment during the observation period. VA configurations were similar across the two groups, with radiocephalic AVFs being the most common (61% in both groups).

After propensity score matching, the dataset included 496 patients with ADPKD and 4321 matched controls (non-ADPKD). Their baseline characteristics are presented in Table 2.

**Table 2:** Baseline characteristics of patients after performing propensity score matching

Characteristic	Non-ADPKD Patients (*n*=4321)	Patients with ADPKD (*n*=496)	*P* Value
Age, mean years (SD)	61.8 (14.9)	61.2 (12.2)	0.001
**Sex, No. (%)**			0.11
Male	2549 (59.0)	274 (55.2)	
Female	1772 (41.0)	222 (44.8)	
**Anticoagulation, No. (%)**			0.48
Yes	765 (17.7)	81 (16.3)	
No	887 (20.5)	113 (22.8)	
Unknown	2669 (61.8)	302 (60.9)	
**Cause of renal failure, No. (%)**			0.001
ADPKD	0 (0.0)	496 (100)	
GN	718 (16.6)		
Pyelonephritis	151 (3.5)		
Hypertension	734 (17.0)		
Renovascular disease	27 (0.6)		
Diabetic nephropathy	1275 (29.5)		
Unknown uremia	470 (10.9)		
Other	946 (21.9)		
**Type of VA, No. (%)**			0.30
Radiocephalic AVF	2566 (59.4)	303 (61.1)	
Brachiocephalic AVF	941 (21.8)	92 (18.5)	
Brachiobasilic AVF	169 (3.9)	22 (4.4)	
Forearm AVG	52 (1.2)	5 (1.0)	
Upper arm graft	494 (11.4)	56 (11.3)	
Forearm AVF other	99 (2.3)	18 (3.6)	
**Phase at VA surgery, No. (%)**			0.001
Preemptive	1786 (41.6)	256 (51.6)	
On dialysis	2270 (52.5)	190 (38.3)	
Kidney transplant	166 (3.8)	35 (7.1)	
Unknown	99 (2.3)	15 (3.0)	
**Comorbidities, No. (%)**			
Hematologic malignancy	84 (1.9)	1 (0.2)	0.02
Skin malignancy	30 (0.7)	1 (0.2)	0.41
Other malignancy	384 (8.1)	21 (4.2)	0.01
Diabetes mellitus	1684 (39.0)	30 (6.0)	0.001
Ischemic heart disease	648 (15.0)	31 (6.3)	0.001
Treated for hypertension	2729 (63.2)	306 (61.7)	0.78
Cardiovascular disease	285 (6.6)	32 (6.5)	0.96
Peripheral vascular disease	277 (6.4)	10 (2.0)	0.001

The list of causes of renal failure listed as “other” is presented in Supplemental Table 1. Dialysis treatment encompasses both peritoneal dialysis and hemodialysis. ADPKD, autosomal dominant polycystic kidney disease; AVF, arteriovenous fistula; AVG, arteriovenous graft; VA, vascular access.

### Functional Start of the VA

Patients with ADPKD had a similar proportion of absence of a recorded functional start as non-ADPKD patients (20.0% versus 19.6%, *P* = 0.83). Although nonsignificant, slightly more patients with ADPKD had an intervention-free functional start (65.1% versus 62.8%, *P* = 0.13, Table 3). There was no difference in the percentage of patients with assisted maturation (14.9% of patients with ADPKD and 17.6% of patients with non-ADPKD *P* = 0.32). There was a weak correlation between the time from VA surgery to first cannulation/functional use for hemodialysis and the duration until loss of primary patency: Pearson correlation coefficient was 0.20/0.17 in our patients with ADPKD and 0.16/0.15 for non-ADPKD patients (*P* < 0.001 in all analyses).

**Table 3:** Maturation outcomes and time until use of the vascular access

Functional Start Parameters	Non-ADPKD Patients	ADPKD Patients	*P* Value
No functional start, No. (%)	845 (19.6)	99 (20.0)	0.83
Intervention-free functional start, No. (%)	2714 (62.8)	323 (65.1)	0.13
Mean time from VA surgery to functional start in days (95% CI)	117 (110 to 124)	122 (102 to 143)	0.01
Mean time from first cannulation to functional start in days (95% CI)	28 (25 to 32)	37 (25 to 49)	0.02

Time until functional start and between first cannulation and functional start was analyzed in the subset of patients who already received dialysis when undergoing vascular access surgery. *N*=1797 non-ADPKD patients (79%) and 141 patients with autosomal dominant polycystic kidney disease (90%) who were on dialysis when receiving their vascular access and had a functional start of the newly created vascular access. Significance was assessed with the Pearson chi-square test. ADPKD, autosomal dominant polycystic kidney disease; VA, vascular access: arteriovenous fistulas and grafts.

Subsequently, we analyzed the subgroup of patients who were already on dialysis when receiving an AVF/AVG. Patients with ADPKD already on dialysis when receiving a VA had a longer period between surgery and functional start of the VA than controls (non-ADPKD) (122 versus 117 days, *P* = 0.01, Table 3). They also experienced a longer interval between first cannulation and use of the VA (37 versus 28 days *P* = 0.02, Table 3) compared with other patients with ESKD who were on dialysis.

### First Interventions Ending Primary Patency

Half of all patients underwent one VA-related intervention at minimum during follow-up (51.8% of patients with ADPKD versus 52.1% in controls, *P* = 0.91, Table 4). Around 3% of all primary interventions was performed to reduce flow (3.1% of primary interventions in patients with ADPKD and 2.9% in controls, *P* = 0.71). Patients with ADPKD had more first interventions to re-establish flow when compared with non-ADPKD controls (49.4% versus 41.9%, *P* = 0.02), while more non-ADPKD patients received interventions to maintain VA patency (53.6% versus 46.7% of patients with ADPKD, *P* = 0.04), other interventions were hybrid or not defined. Overall, patients with ADPKD had fewer interventions per year of functional patency than non-ADPKD controls (1.15±0.13 SEM, versus 1.30±0.05, *P* < 0.001).

**Table 4:** Patency rates at month 3, 1, and 3 years

Patency	3 mo		1 yr		3 yr	
	Non-ADPKD	ADPKD	Non-ADPKD	ADPKD	Non-ADPKD	ADPKD
Primary	77.5%	79.8%	41.2%	45.6%	17.8%	21.4%
Postcannulation primary	72.1%	75.3%	45.2%	48.0%	20.1%	21.6%
Secondary	90.5%	90.5%	72.7%	75.2%	40.3%	42.9%
Functional	92.8%	94.3%	76.3%	79.0%	40.1%	42.2%

Primary, postcannulation primary, secondary, and functional patency rates at different time intervals: 3 months, 1, and 3 years, expressed as percentage of all vascular accesses within the analysis subset. ADPKD, autosomal dominant polycystic kidney disease.

### VA Patency Did Not Differ between Patients with ADPKD and Non-ADPKD Patients

**Figure 2:**
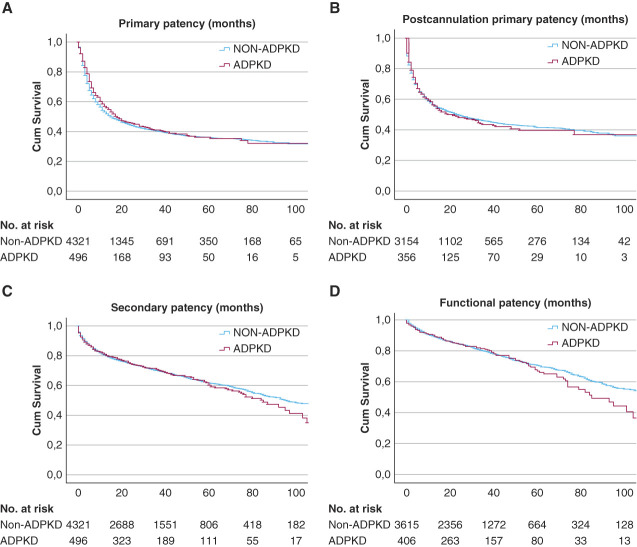
**Survival curves of patency outcomes.** Primary (A), postcannulation (B), secondary (C), and functional patency (D) in months for arteriovenous fistulas and grafts in patients with ADPKD and controls.

The median follow-up time in the ADPKD group was 30 months and 28 months in the non-ADPKD group (*P* < 0.001). Table 4 presents the patency rates at 3 months, 1 year, and 3 years in patients with ADPKD and non-ADPKD controls, which were higher in patients with ADPKD. Patients with ADPKD did not have different VA outcomes compared with non-ADPKD controls (Figure 2): The median primary patency of patients with ADPKD was 18 versus 15 months in non-ADPKD controls (*P* = 0.33). Postcannulation primary patency was 21 versus 24 months (*P* = 0.80), respectively. Long-term patency was also comparable: Secondary patency was median 84 months in patients with ADPKD and 95 months in non-ADPKD controls (*P* = 0.40) and functional patency 85 versus 112 months (*P* = 0.15).

### End of Use of the VA

More patients with ADPKD received a donor kidney (19.4% versus 11.3%, *P* = 0.001, Table 5), and more VAs were abandoned, although nonsignificant (36.5% versus 33.3%, *P* = 0.15). Ligation of the VA because of complications, such as steal syndrome, high flow, bleeding, or aneurysm formation of the VA, was more common among patients with ADPKD (10.5% versus 7.7%, *P* = 0.03).

**Table 5:** Vascular access interventions and end of use

Interventions and Abandonment	Non-ADPKD	ADPKD	*P* Value
Received a first intervention, No. (%)	2251 (52.1)	257 (51.8)	0.91
Primary intervention to maintain patency, No. (%)	1206 (53.6)	120 (46.7)	0.04
Primary intervention to re-establish patency, No. (%)	944 (41.9)	127 (49.4)	0.02
VA is abandoned, No. (%)	1437 (33.3)	181 (36.5)	0.15
Received a transplant kidney, No. (%)	489 (11.3)	96 (19.4)	0.001
VA is ligated, No. (%)	333 (7.7)	52 (10.5)	0.03

Ligation can occur because of aneurysm formation of the vascular access, bleeding of the vascular access, high flow, or steal syndrome. ADPKD, autosomal dominant polycystic kidney disease; VA, vascular access: arteriovenous fistulas and grafts.

## Discussion

In this study, we hypothesized that ADPKD may affect VA patency. However, patency of AVFs and AVGs in patients with ADPKD did not differ compared with patients with other causes of ESKD. We did not observe any significant differences in short-term VA patency. Approximately 20% of all AVFs/AVGs did not have a functional start, comparable with outcomes in other cohorts.^20–22^ Although not statistically significant, mean primary patency seemed to be higher in patients with ADPKD, and more patients with ADPKD achieved an intervention-free functional start of the VA. However, after approximately 5 years, secondary and functional patency rates seemed worse in patients with ADPKD.

A previous study in Taiwan showed that only in the long term (1–10 years after VA surgery), incidence rates of AVF/AVG dysfunction were higher in patients with ADPKD.^15^ However, they did not elaborate on the functionality or patency rates of AVFs/AVGs in patients with ADPKD. By using the Swedish Renal Registry, we aimed to create a representative cohort of patients with ADPKD in Europe and add to the previous findings from Taiwan by generating more data on the functionality of VAs created in patients with ADPKD. The SRR is a nationwide quality register in which loss of follow-up only occurs when patients emigrate and thus enables analysis of large patient groups. As ADPKD affects the vasculature due to ADPKD's underlying genetic mechanisms, we aimed to compare AVF/AVG patency in patients with and without ADPKD.

Mutations in the PKD1 or PKD2 gene account for approximately 93% of patients with polycystic kidney disease, and the remaining 7% of patients with cystic kidneys have either an undiagnosed mutation or a mutation in genes that are involved in polycystic kidney disease protein trafficking or biogenesis (GANAB, DNAJB11, IFT140, or ALG9).^23–28^ PKD1 and PKD2 are expressed in endothelial cells and vascular smooth muscle cells and encode Polycystin proteins-1 and -2.^29^ Polycystin-1 and -2 help maintain vessel wall integrity and regulate extracellular matrix homeostasis.^30–32^ The Polycystin proteins together form an ion channel complex regulating calcium influx and are involved in sensing mechanotransduction and fluid flow.^33,34^ Thus, it could be hypothesized that vascular remodeling occurs differently in patients with ADPKD compared with others receiving an AVF or AVG, as shown by their tendency to develop intracranial and abdominal aneurysms^9–12^ and in AVFs specifically.^14^ We did observe increased frequency of VA ligation in patients with ADPKD; however, the reason for ligation was not registered, so we cannot report the numbers on aneurysm development and required interventions. Nonetheless, because ligation is performed after complications such as high flow, aneurysm formation, excessive bleeding of the VA or steal syndrome, this might indicate that patients with ADPKD have altered vascular remodeling that facilitates increase in blood flow more adequately than other underlying reasons for ESKD. In both groups, time until functional patency was comparable with the reported 3.5 months in a systematic review by Bylsma *et al.* covering 50 articles reporting time until maturation.^35^

A large fraction of VAs in our dataset are created preemptively. Because there are national recommendations in place to screen for CKD in patients with increased risk—*e.g*., individuals with diabetes mellitus, hypertension, or cardiovascular disease—and equal access to health care, patients with CKD are referred to the nephrologist early on. More than 80% of the patients on dialysis are already registered in the SRR before initiation of dialysis, and preemptive VAs are seen as a quality indicator for adequate patient monitoring and care. It is thought that fistula creation before hemodialysis initiation does not affect primary or secondary patency.^36^ Moreover, because of the hereditary aspect of the disease, most patients with ADPKD are known and monitored early on by nephrologists. This enables timely access planning or opting for a (preemptive) transplant. In our cohort, more patients with ADPKD received their VA earlier on, before requiring RRT, and were thus less uremic at the initiation of hemodialysis, requiring fewer dialysis sessions or starting at a later time point. This could explain the 5-day longer interval from surgery to functional initiation of the VA in patients with ADPKD, despite fewer patients requiring an intervention to start using the VA. Patients from the United States using a central venous cathether when receiving an AVF/AVG showed an association between the number of interventions needed to achieve maturation and the risk of losing primary patency^37^; however, we observed a weak correlation between the time until first cannulation or functional start and the duration until loss of primary patency in our dataset for both patients with ADPKD and non-ADPKD patients receiving an AVF/AVG.

Compared with other patients with non-diabetic ESKD and patients with non-ADPKD diseases on hemodialysis, patients with ADPKD have increased survival rates and better overall health,^38,39^ which are also reflected by the low prevalence of diabetes mellitus and peripheral vascular disease in our ADPKD cohort. This could counteract some of the ADPKD-related intrinsic vascular changes affecting VA maturation. Timely diagnosis and referral to the nephrologist also supports planning for transplantation, so that relatively healthy patients with ADPKD transitioned from hemodialysis to transplantation while sicker patients remained on hemodialysis, and this may be a selection bias negatively affecting long-term secondary and functional patency analysis.

To minimize loss of power, we performed one to many propensity score matching, with a caliper of 0.1. This resulted in fuzzy matching regarding the phase of kidney failure when the patients received their AVF/AVG. The large number of preemptive fistulas created in patients with ADPKD compared with controls, where half of the patients already received dialysis, could explain why more patients with ADPKD underwent first VA interventions to re-establish patency. Patients receiving dialysis visit the hospital about three times a week, where the new VA is monitored by the dialysis nurse while a preemptive fistula or graft undergoes clinical evaluation approximately 4–6 weeks after surgery, using duplex ultrasound. The delayed monitoring and more sensitive evaluation might result in both prolonged progression of stenosis, quick detection of a potential stenosis, and more severe interventions, such as thrombectomy or anastomosis revision to ensure functionality of the VA for future use. Overall, patients with ADPKD did have less interventions per functional patency years than controls. The large number of patients and long follow-up in the Swedish Renal Registry allows for subgroup analysis, which can help answer pressing research questions in the field of VA, such as whether VAs created in pre-ESKD have differential outcomes and interventions compared with VAs created when the patient already undergoes RRT. Future research should investigate the effect of phase of kidney failure on VA functionality.

The prevalence of patients with ADPKD with an AVF/AVG in our dataset was 8%. This is comparable with the prevalence of patients with ADPKD on RRT in the SRR, which ranged from 10% to 13% over the past decade, similar to the prevalence reported in Europe.^7^ Only one third of patients with non-ADPKD receiving an AVF/AVG were female. It is known that although CKD is more prevalent in women, kidney failure progresses more quickly to ESKD in men than women.^40^ Biological aspects could underlie the more rapid progression in men, but also personal preferences for RRT modality can explain why more men proceed to hemodialysis treatment.^40,41^ The sex discrepancy in progression to ESKD is also present in patients with ADPKD,^8,42–44^ although to a much lesser extent when compared with other types of kidney failure.

There were several factors limiting our study. First, not all complications are registered in the SRR because they are registered only when resulting in an intervention. This might result in underrepresentation of overall complications. Second, we cannot report the size of the AVF or the vessels that were used for VA surgery because data on pre- and postoperative diameters of the artery, vein, and AVF are not reported in the registry. Third, we can only report on the use of anticoagulation (yes/no), but not the exact medication that was given.

In conclusion, no significant differences in AVF/AVG outcomes were observed in patients with ADPKD compared with patients with other underlying causes of ESKD. This suggests that clinical recommendations for differential monitoring and treatment of VA access is not warranted for patients with ADPKD.

## Supplementary Material

## Data Availability

Partial restrictions to the data and/or materials apply. The data originates from the Swedish Renal Registry and contains personal patient information. Data can be retrieved from the registry upon request and after approval by the Regional Ethical Review Board.

## Supplemental Material

Supplemental Figure 1. Histogram of the propensity scores before and after matching.

Supplemental Table 1. List of renal diseases classified as “other” in Tables 1 and 2.
