# Supplementary material for: Vascular Access Outcomes in Patients with Autosomal Dominant Polycystic Kidney Disease
Source: Kidney360. 2024 May 1;5(6):877–85. doi: 10.34067/KID.0000000000000453 (PMC11219118; doi:10.34067/KID.0000000000000453)
Supplement: Supplementary file 1 [file kidney360-5-877-s001.pdf]

ASN Journal Disclosure Form

As per ASN journal policy, I have disclosed any financial relationship or commitment held by myself and/or my spouse/partner in the past 36 months as included below. I have listed my Current Employer below to indicate there is a relationship requiring disclosure. If no relationship exists, my Current Employer is not listed.

S. Josemans has nothing to disclose.

I understand that the information above will be published within the journal article, if accepted, and that failure to comply and/or to accurately and completely report the potential financial conflicts of interest could lead to the following: 1) Prior to publication, article rejection, or 2) Post-publication, sanctions ranging from, but not limited to, issuing a correction, reporting the inaccurate information to the authors' institution, banning authors from submitting work to ASN journals for varying lengths of time, and/or retraction of the published work.

Name: Sabine H. Josemans
Manuscript ID: K360-2024-000037R1
Manuscript Title: Vascular access outcomes in patients with autosomal dominant polycystic kidney disease (ADPKD)
Date of Completion: April 3, 2024
Disclosure Updated Date: April 3, 2024

ASN
American Society of Nephrology
JASN
Journal of the American Society of Nephrology
KIDNEY360
Accessing Our World From Every Angle
CJASN
Clinical Journal of the American Society of Nephrology

ASN Journal Disclosure Form

As per ASN journal policy, I have disclosed any financial relationship or commitment held by myself and/or my spouse/partner in the past 36 months as included below. I have listed my Current Employer below to indicate there is a relationship requiring disclosure. If no relationship exists, my Current Employer is not listed.

S. Laboyrie reports the following:
Employer: Leiden University Medical Center (LUMC)

I understand that the information above will be published within the journal article, if accepted, and that failure to comply and/or to accurately and completely report the potential financial conflicts of interest could lead to the following: 1) Prior to publication, article rejection, or 2) Post-publication, sanctions ranging from, but not limited to, issuing a correction, reporting the inaccurate information to the authors' institution, banning authors from submitting work to ASN journals for varying lengths of time, and/or retraction of the published work.

Name: Suzanne Laboyrie
Manuscript ID: K360-2024-000037R1
Manuscript Title: Vascular access outcomes in patients with autosomal dominant polycystic kidney disease (ADPKD)
Date of Completion: April 3, 2024
Disclosure Updated Date: May 23, 2023

ASN American Society of Nephrology JASN Journal of the American Society of Nephrology KIDNEY360 Accessing Our World From Every Angle CJASN Clinical Journal of the American Society of Nephrology

ASN Journal Disclosure Form

As per ASN journal policy, I have disclosed any financial relationships or commitments I have held in the past 36 months as included below. I have listed my Current Employer below to indicate there is a relationship requiring disclosure. If no relationship exists, my Current Employer is not listed.

J. Rotmans reports the following:
Employer: LUMC; Consultancy: Xeltis BV; Ownership Interest: XS Innovations; Research Funding: AstraZeneca; Advisory or Leadership Role: president Vascular Access Society; Advisory Board Nextkidney; and Other Interests or Relationships: Chair Thematic Working Group Vascular Tissue Engineering at TERMIS.

I understand that the information above will be published within the journal article, if accepted, and that failure to comply and/or to accurately and completely report the potential financial conflicts of interest could lead to the following: 1) Prior to publication, article rejection, or 2) Post-publication, sanctions ranging from, but not limited to, issuing a correction, reporting the inaccurate information to the authors' institution, banning authors from submitting work to ASN journals for varying lengths of time, and/or retraction of the published work.

Name: Joris I. Rotmans
Manuscript ID: K360-2024-000037R1
Manuscript Title: Vascular access outcomes in patients with autosomal dominant polycystic kidney disease
Date of Completion: April 18, 2024
Disclosure Updated Date: March 25, 2024

ASN American Society of Nephrology
JASN Journal of the American Society of Nephrology
KIDNEY360 Accessing Our World From Every Angle
CJASN Clinical Journal of the American Society of Nephrology

ASN Journal Disclosure Form

As per ASN journal policy, I have disclosed any financial relationship or commitment held by myself and/or my spouse/partner in the past 36 months as included below. I have listed my Current Employer below to indicate there is a relationship requiring disclosure. If no relationship exists, my Current Employer is not listed.

B. Sigvant has nothing to disclose.

I understand that the information above will be published within the journal article, if accepted, and that failure to comply and/or to accurately and completely report the potential financial conflicts of interest could lead to the following: 1) Prior to publication, article rejection, or 2) Post-publication, sanctions ranging from, but not limited to, issuing a correction, reporting the inaccurate information to the authors' institution, banning authors from submitting work to ASN journals for varying lengths of time, and/or retraction of the published work.

Name: Birgitta M Sigvant
Manuscript ID: K360-2024-000037R1
Manuscript Title: ascular access outcomes in patients with autosomal dominant polycystic kidney disease (ADPKD
Date of Completion: April 3, 2024
Disclosure Updated Date: April 3, 2024

ASN American Society of Nephrology
JASN Journal of the American Society of Nephrology
KIDNEY360 Accessing Our World From Every Angle
CJASN Clinical Journal of the American Society of Nephrology

ASN Journal Disclosure Form

As per ASN journal policy, I have disclosed any financial relationship or commitment held by myself and/or my spouse/partner in the past 36 months as included below. I have listed my Current Employer below to indicate there is a relationship requiring disclosure. If no relationship exists, my Current Employer is not listed.

M. Svensson reports the following:
Employer: Uppsala University, Uppsala, Sweden; and Honoraria: Amgen, AstraZeneca, Boehringer Ingelheim, GSK, NovoNordisk.

I understand that the information above will be published within the journal article, if accepted, and that failure to comply and/or to accurately and completely report the potential financial conflicts of interest could lead to the following: 1) Prior to publication, article rejection, or 2) Post-publication, sanctions ranging from, but not limited to, issuing a correction, reporting the inaccurate information to the authors' institution, banning authors from submitting work to ASN journals for varying lengths of time, and/or retraction of the published work.

Name: Maria K. Svensson
Manuscript ID: K360-2024-000037R1
Manuscript Title: Vascular access outcomes in patients with autosomal dominant polycystic kidney disease (ADPKD)
Date of Completion: April 2, 2024
Disclosure Updated Date: May 16, 2023

ASN Journal Disclosure Form

As per ASN journal policy, I have disclosed any financial relationship or commitment held by myself and/or my spouse/partner in the past 36 months as included below. I have listed my Current Employer below to indicate there is a relationship requiring disclosure. If no relationship exists, my Current Employer is not listed.

G. Welander reports the following:
Employer: Region Värmland Centralsjukhuset

I understand that the information above will be published within the journal article, if accepted, and that failure to comply and/or to accurately and completely report the potential financial conflicts of interest could lead to the following: 1) Prior to publication, article rejection, or 2) Post-publication, sanctions ranging from, but not limited to, issuing a correction, reporting the inaccurate information to the authors' institution, banning authors from submitting work to ASN journals for varying lengths of time, and/or retraction of the published work.

Name: Gunilla Welander
Manuscript ID: K360-2024-000037R1
Manuscript Title: "Vascular access outcomes in patients with autosomal dominant polycystic kidney disease (ADPKD),"
Date of Completion: April 4, 2024
Disclosure Updated Date: April 4, 2024
