# Supplementary material for: Vascular Access Outcomes in Patients with Autosomal Dominant Polycystic Kidney Disease
Source: Kidney360. 2024 May 1;5(6):877–85. doi: 10.34067/KID.0000000000000453 (PMC11219118; doi:10.34067/KID.0000000000000453)
Supplement: Supplementary file 2 [file kidney360-5-877-s002.pdf]

Supplemental data

Supplementary figure 1: Histogram of the propensity scores before and after matching

Supplementary table 1: List of renal diseases classified as ‘other’ in Table 1 and 2.

Supplementary figure 1: Histogram of the propensity scores before and after matching

Propensity scores of the ADPKD group remained the same before (A) and after (B) propensity score matching: all patients were included in the final dataset. Individuals in the non-ADPKD group had a different distribution before the matching (C), but similar to the ADPKD group after propensity score matching (D).

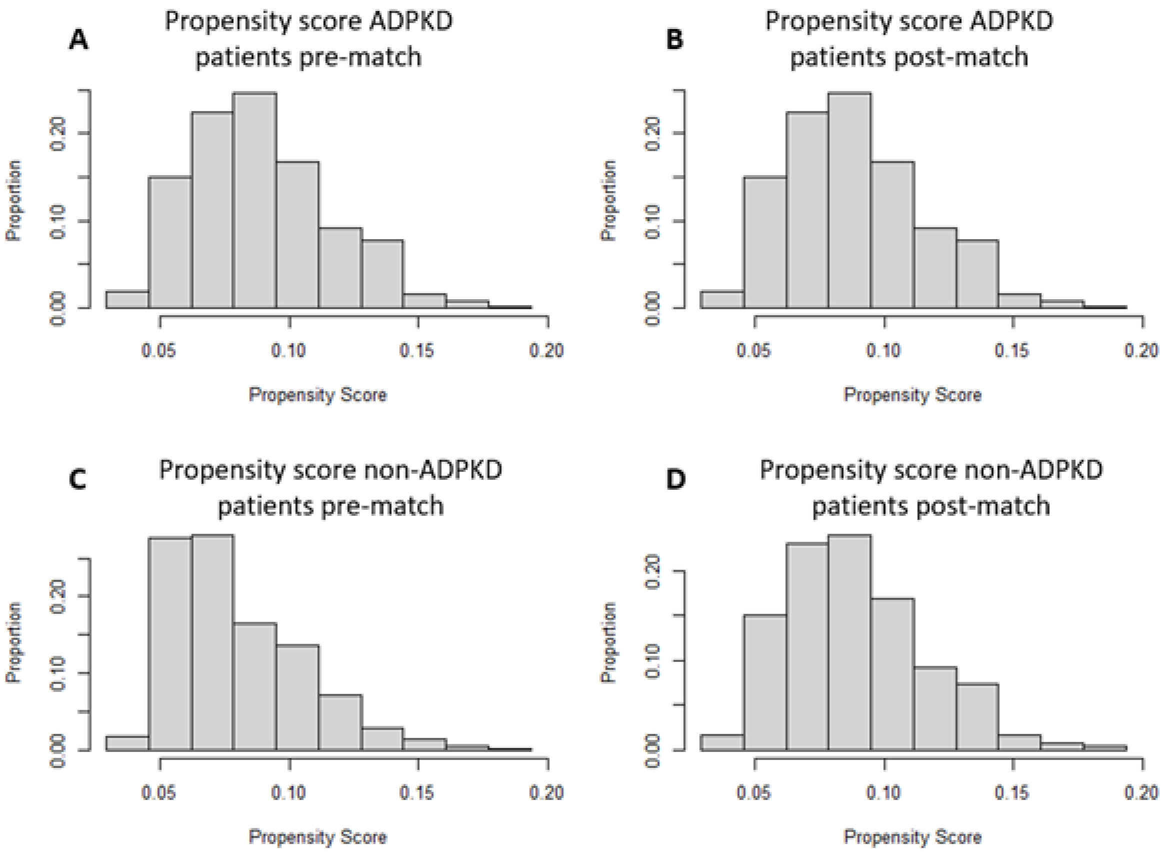

Supplementary table 1: List of renal diseases classified as ‘other’ in Table 1 and 2.

EDTA PRD code	ERA-EDTA Primary Renal Diagnosis (PRD) Term
**1074**	Denys-Drash syndrome
**1279**	Familial focal segmental glomerulosclerosis (FSGS) - autosomal recessive - no histology
**1298**	Familial focal segmental glomerulosclerosis (FSGS) - autosomal dominant - no histology
**1396**	Systemic vasculitis - ANCA positive - no histology
**1401**	Granulomatosis with polyangiitis - no histology
**1417**	Granulomatosis with polyangiitis - histologically proven
**1429**	Microscopic polyangiitis - histologically proven
**1438**	Churg-Strauss syndrome - no histology
**1440**	Churg-Strauss syndrome - histologically proven
**1455**	Polyarteritis nodosa
**1464**	Anti-Glomerular basement membrane (GBM) disease / Goodpasture's syndrome - no histology
**1472**	Anti-Glomerular basement membrane (GBM) disease / Goodpasture's syndrome - histologically proven
**1486**	Systemic lupus erythematosus / nephritis - no histology
**1493**	Systemic lupus erythematosus / nephritis - histologically proven
**1504**	Henoch-Schönlein purpura / nephritis - no histology
**1515**	Henoch-Schönlein purpura / nephritis - histologically proven
**1527**	Renal scleroderma / systemic sclerosis - no histology
**1536**	Renal scleroderma / systemic sclerosis - histologically proven
**1543**	Essential mixed cryoglobulinaemia - no histology
**1558**	Essential mixed cryoglobulinaemia - histologically proven
**1562**	Cryoglobulinaemia secondary to hepatitis C - no histology
**1570**	Cryoglobulinaemia secondary to hepatitis C - histologically proven
**1589**	Cryoglobulinaemia secondary to systemic disease - no histology
**1591**	Cryoglobulinaemia secondary to systemic disease - histologically proven
**1625**	Congenital dysplasia / hypoplasia
**1639**	Multicystic dysplastic kidneys
**1641**	Dysplasia due to fetal ACE-inhibitor exposure
**1656**	Glomerulocystic disease
**1660**	Congenital pelvi-ureteric junction obstruction
**1673**	Congenital vesico-ureteric junction obstruction
**1687**	Posterior urethral valves
**1694**	Syndrome of agenesis of abdominal muscles - prune belly syndrome
**1706**	Congenital neurogenic bladder
**1710**	Bladder exstrophy
**1723**	Megacystis-megaureter
**1734**	Oligomeganephronia
**1747**	Renal papillary necrosis - cause unknown
**1845**	Calcium oxalate urolithiasis

**1850**	Enteric hyperoxaluria
**1878**	Uric acid urolithiasis
**1884**	Tubulointerstitial nephritis - no histology
**1897**	Tubulointerstitial nephritis - histologically proven
**1907**	Familial interstitial nephropathy - no histology
**1911**	Familial interstitial nephropathy - histologically proven
**1924**	Tubulointerstitial nephritis associated with autoimmune disease - no histology
**1930**	Tubulointerstitial nephritis associated with autoimmune disease - histologically proven
**1948**	Tubulointerstitial nephritis with uveitis (TINU) - no histology
**1953**	Tubulointerstitial nephritis with uveitis (TINU) - histologically proven
**1969**	Renal sarcoidosis - no histology
**1976**	Renal sarcoidosis - histologically proven
**1982**	Aristolochic acid nephropathy (Balkan / Chinese herb / endemic nephropathy) - no histology
**1995**	Aristolochic acid nephropathy (Balkan / Chinese herb / endemic nephropathy) - histologically proven
**2005**	Drug-induced tubulointerstitial nephritis - no histology
**2014**	Drug-induced tubulointerstitial nephritis - histologically proven
**2022**	Nephropathy due to analgesic drugs - no histology
**2033**	Nephropathy due to analgesic drugs - histologically proven
**2046**	Nephropathy due to ciclosporin - no histology
**2051**	Nephropathy due to ciclosporin - histologically proven
**2067**	Nephropathy due to tacrolimus - no histology
**2079**	Nephropathy due to tacrolimus - histologically proven
**2080**	Nephropathy due to aminoglycosides - no histology
**2098**	Nephropathy due to aminoglycosides - histologically proven
**2108**	Nephropathy due to amphotericin - no histology
**2112**	Nephropathy due to amphotericin - histologically proven
**2120**	Nephropathy due to cisplatin - no histology
**2131**	Nephropathy due to cisplatin - histologically proven
**2149**	Nephropathy due to lithium - no histology
**2154**	Nephropathy due to lithium - histologically proven
**2165**	Lead induced nephropathy - no histology
**2177**	Lead induced nephropathy - histologically proven
**2183**	Acute urate nephropathy - no histology
**2196**	Acute urate nephropathy - histologically proven
**2203**	Chronic urate nephropathy - histologically proven
**2219**	Radiation nephritis
**2226**	Renal / perinephric abscess
**2235**	Renal tuberculosis
**2242**	Leptospirosis
**2257**	Hantavirus nephropathy
**2261**	Xanthogranulomatous pyelonephritis
**2274**	Nephropathy related to HIV - no histology

**2288**	Nephropathy related to HIV - histologically proven
**2290**	Schistosomiasis
**2300**	Other specific infection
**2407**	Ischaemic nephropathy - no histology
**2411**	Ischaemic nephropathy / microvascular disease - histologically proven
**2430**	Atheroembolic renal disease - no histology
**2448**	Atheroembolic renal disease - histologically proven
**2476**	Renal vein thrombosis
**2482**	Cardiorenal syndrome
**2495**	Hepatorenal syndrome
**2509**	Renal amyloidosis
**2513**	AA amyloid secondary to chronic inflammation
**2521**	AL amyloid secondary to plasma cell dyscrasia
**2532**	Familial amyloid secondary to protein mutations - no histology
**2545**	Familial amyloid secondary to protein mutations - histologically proven
**2550**	Familial AA amyloid secondary to familial Mediterranean fever / TRAPS (Hibernian fever) - no histology
**2566**	Familial AA amyloid secondary to familial Mediterranean fever / TRAPS (Hibernian fever) - histologically proven
**2578**	Myeloma kidney - no histology
**2584**	Myeloma cast nephropathy - histologically proven
**2597**	Light chain deposition disease
**2606**	Immunotactoid / fibrillary nephropathy
**2610**	Haemolytic uraemic syndrome (HUS) - diarrhoea associated
**2623**	Atypical haemolytic uraemic syndrome (HUS) - diarrhoea negative
**2634**	Thrombotic thrombocytopenic purpura (TTP)
**2647**	Haemolytic uraemic syndrome (HUS) secondary to systemic disease
**2652**	Congenital haemolytic uraemic syndrome (HUS)
**2668**	Familial haemolytic uraemic syndrome (HUS)
**2675**	Familial thrombotic thrombocytopenic purpura (TTP)
**2681**	Nephropathy due to pre-eclampsia / eclampsia
**2699**	Sickle cell nephropathy - no histology
**2702**	Sickle cell nephropathy - histologically proven
**2741**	Autosomal recessive (AR) polycystic kidney disease
**2756**	Alport syndrome - no histology
**2760**	Alport syndrome - histologically proven
**2773**	Benign familial haematuria
**2787**	Thin basement membrane disease
**2794**	Cystic kidney disease
**2804**	Medullary cystic kidney disease type I
**2815**	Medullary cystic kidney disease type II
**2827**	Uromodulin-associated nephropathy (familial juvenile hyperuricaemic nephropathy)
**2836**	Nephronophthisis

**2843**	Nephronophthisis - type 1 (juvenile type)
**2858**	Nephronophthisis - type 2 (infantile type)
**2862**	Nephronophthisis - type 3 (adolescent type)
**2870**	Nephronophthisis - type 4 (juvenile type)
**2889**	Nephronophthisis - type 5
**2891**	Nephronophthisis - type 6
**2901**	Primary Fanconi syndrome
**2917**	Tubular disorder as part of inherited metabolic diseases
**2929**	Dent disease
**2938**	Lowe syndrome (oculocerebrorenal syndrome)
**2940**	Inherited aminoaciduria
**2955**	Cystinuria
**2964**	Cystinosis
**2972**	Inherited renal glycosuria
**2986**	Hypophosphataemic rickets X-linked (XL)
**2993**	Hypophosphataemic rickets autosomal recessive (AR)
**3000**	Primary renal tubular acidosis (RTA)
**3016**	Proximal renal tubular acidosis (RTA) - type II
**3028**	Distal renal tubular acidosis (RTA) - type I
**3037**	Distal renal tubular acidosis with sensorineural deafness - gene mutations
**3044**	Nephrogenic diabetes insipidus
**3059**	Lesch Nyhan syndrome - hypoxanthine guanine phosphoribosyl transferase deficiency
**3063**	Phosphoribosyl pyrophosphate synthetase (PRPPS) superactivity
**3071**	Alagille syndrome
**3085**	Bartter syndrome
**3092**	Gitelman syndrome
**3102**	Liddle syndrome
**3118**	Apparent mineralocorticoid excess
**3125**	Glucocorticoid suppressible hyperaldosteronism
**3139**	Inherited / genetic diabetes mellitus type II
**3141**	Pseudohypoaldosteronism type 1
**3156**	Pseudohypoaldosteronism type 2 (Gordon syndrome)
**3160**	Familial hypocalciuric hypercalcaemia
**3173**	Familial hypercalciuric hypocalcaemia
**3187**	Familial hypomagnesaemia
**3194**	Primary hyperoxaluria
**3207**	Primary hyperoxaluria type I
**3211**	Primary hyperoxaluria type II
**3224**	Fabry disease - no histology
**3230**	Fabry disease - histologically proven
**3248**	Xanthinuria
**3253**	Nail-patella syndrome
**3269**	Rubinstein-Taybi syndrome

**3276**	Tuberous sclerosis
**3282**	Von Hippel-Lindau disease
**3295**	Medullary sponge kidneys
**3305**	Horse-shoe kidney
**3314**	Frasier syndrome
**3322**	Branchio-oto-renal syndrome
**3333**	Williams syndrome
**3346**	Townes-Brocks syndrome
**3351**	Lawrence-Moon-Biedl / Bardet-Biedl syndrome
**3367**	Mitochondrial cytopathy
**3379**	Familial nephropathy
**3380**	Acute kidney injury
**3398**	Acute kidney injury due to hypovolaemia
**3403**	Acute kidney injury due to circulatory failure
**3419**	Acute kidney injury due to sepsis
**3426**	Acute kidney injury due to rhabdomyolysis
**3435**	Acute kidney injury due to nephrotoxicity
**3442**	Acute cortical necrosis
**3461**	Kidney tumour
**3474**	Renal cell carcinoma - histologically proven
**3488**	Transitional cell carcinoma - histologically proven
**3490**	Wilms tumour - histologically proven
**3501**	Mesoblastic nephroma - histologically proven
**3517**	Single kidney identified in adulthood
**3529**	Chronic kidney disease (CKD) / chronic renal failure (CRF) caused by tumour nephrectomy
**3538**	Chronic kidney disease (CKD) / chronic renal failure (CRF) due to traumatic loss of kidney
**3731**	Primary hyperoxaluria type III
